# Antidiabetic Compounds in* Syzygium cumini* Decoction and Ready to Serve Herbal Drink

**DOI:** 10.1155/2017/1083589

**Published:** 2017-05-09

**Authors:** P. R. D. Perera, S. Ekanayake, K. K. D. S. Ranaweera

**Affiliations:** ^1^Department of Food Science and Technology, Faculty of Applied Sciences, University of Sri Jayewardenepura, Nugegoda, Sri Lanka; ^2^Department of Biochemistry, Faculty of Medical Sciences, University of Sri Jayewardenepura, Nugegoda, Sri Lanka

## Abstract

Herbal beverages with desirable sensory attributes are an ideal way to offer consumers with phytochemicals having specific health promoting functionalities.* Syzygium cumini* bark decoction is used in treating diabetes mellitus in Ayurveda medicine. This work attempted to prove the presence of antidiabetic compounds in the* S. cumini* decoction and the ready to serve (RTS) herbal drink developed using the decoction. Activity guided fractionation of the decoction of the* S. cumini* was carried out by sequential extraction with organic solvents of different polarities. Thin Layer Chromatography (TLC) with authentic compounds and HPLC were performed for identification and confirmation of the compounds in the decoction and the RTS herbal drink. Presence of gallic and ellagic acids in the decoction and RTS herbal drink was proven and confirmed with HPLC. The LC UV-VIS spectra of the two compounds were identical with the corresponding spectra of the library. Further, gallic acid and umbelliferone were determined as the active compounds in the decoction by TLC and were confirmed by cochromatography with authentic compounds. The present investigation confirmed the presence of gallic acid, ellagic acid, and umbelliferone which are proven to have antidiabetic activities in the decoction and the RTS herbal drink prepared with the decoction.

## 1. Introduction

The global prevalence of diabetes mellitus (DM) is rising at an alarming rate with a reported 381 million affected in 2013. It is projected that 592 million among the world population will be afflicted with diabetes by 2035 [1], with the greatest prevalence in Asia and Africa [2]. According to the latest survey one in four people in Sri Lanka are affected by diabetes or prediabetes. The reported prevalence of diabetes among Sri Lankan adults was nearly 11% with 36% of those with diabetes undiagnosed [1]. The high prevalence of diabetes is attributed to a combination of factors such as high calorie diets, low activity life style, and genetic susceptibility [3].

Hyperglycaemia or high blood glucose is the “hallmark” of diabetes which contributes to the pathogenesis and the many complications of diabetes [4]. Long-term DM leads to macrovascular complications such as coronary artery disease, peripheral arterial disease, stroke, and microvascular complications (diabetic nephropathy, neuropathy, embryopathy, and retinopathy) which decrease the quality of life of individuals with diabetes. The key molecular basis of the long-term diabetic complications is the protein glycation which occurs in the body in accelerated rates under chronic hyperglycemic conditions forming nonreversible advanced glycated end-products (AGEs). Glycation is a nonenzymatic reaction between carbonyl group of reducing [i.e., glucose] sugars and free amino group of biological proteins resulting in the formation of AGEs via formation of Schiff bases and Amadori products [5] with further oxidation and generation of excessive amounts of reactive oxygen species [6]. These irreversible, insoluble, florescent compounds form cross-links with and between protein molecules and compromise their physiological functions. A large body of evidence suggests that AGEs are important pathogenic mediators of almost all diabetic complications.

Despite the pharmacotherapy with insulin or oral hypoglycaemic drugs, the recent global interest in medicinal plants used in traditional medicine has escalated as these have been found to have active principles with antiglycation and antioxidant properties. Thus these plants have the therapeutic potential to prevent the many diabetic complications with minimal adverse effects at a lower cost.* Syzygium cumini* (madan, Sinhala) is a tropical tree (Myrtaceae) of which the bark has been widely used in Ayurveda and Indian folk medicine for the treatment of DM [7]. Diabetic rats treated with* S. cumini* bark had shown significant decrease in blood glucose [8] and positive insulin staining in the epithelia cells of the pancreatic duct [9]. Further, the decoction from the bark extract as used in Ayurveda medicine and a ready to serve (RTS) drink prepared from the decoction had high antiglycation and antioxidant potential [10, 11]. Thus the present study was conducted to identify and confirm the presence of antidiabetic compounds in the decoction of* Syzygium cumini *and to study the availability of such compounds in the ready to serve drink prepared using* S. cumini* decoction.

## 2. Materials and Methods

### 2.1. Collection of Plant Material

Bark samples of* S. cumini* were purchased from the traditional herbal market and the identity of the specimen plant material was authenticated by the Botanist at Bandaranaike Memorial Ayurveda Research Institute, Nawinna, Sri Lanka.

### 2.2. Preparation of Plant Material

The dried bark was ground to a fine powder (National, Japan) at room temperature and stored in air tight containers in a refrigerator (4°C) until used for further analyses.

### 2.3. Preparation of the Decoction

The decoction was prepared by boiling the powdered bark sample (60 g or 12 “kalan”) under low heat with 960 mL of water (4 “patha”) until concentrated to 240 mL (1 “patha”) as according to the traditional method practiced in Ayurveda medicine to prepare “kasaya” (decoction). The concentrate was filtered through a thin silk cloth (500 *μ*m) and freeze-dried (Feyela, FDU-1200) or used to prepare the herbal drink.

### 2.4. Preparation of Herbal Drink

The ready to serve herbal drink which had the highest score from the sensory evaluation containing 20 : 80 mL of decoction and 0.01% sucralose was prepared for confirmation of presence of antidiabetic compounds [11]. Hot filling was carried out into sterilized bottles and capped and pasteurized at 80°C for 20 minutes, cooled, and stored at 4°C.

### 2.5. Activity Guided Fractionation

The freeze-dried (Feyela, FDU-1200) sample of the decoction was subjected to sequential extraction with solvents of different polarities (1 : 1). The fractions obtained by extraction with hexane and ethyl acetate were evaporated under reduced pressure at low temperature (40°C) (BUCHI, Switzerland). The final water extract was freeze-dried. All concentrates were tested for their antioxidant and antiglycation potentials [12]. The ethyl acetate and aqueous fractions had the highest antiglycation and antioxidant activities [13] and thus the ethyl acetate fraction was selected for confirmation of presence of antidiabetic compounds.

### 2.6. Separation and Identification of Active Compounds

Ethyl acetate fraction of the decoction was spotted on the silica gel plate with some available standards of well-known antidiabetic activities [14]. These were *β*-sitosterol, apigenin, umbelliferone, 4-4,5,7-trihydroxyflavone, kaempferol, quercetin, caffeic acid, and gallic acid (40 *μ*L of 50 mg/mL). Toluene: ethyl acetate (Sigma-Aldrich, USA) (7 : 3) was used as the mobile phase. The plate was developed in a saturated glass chamber with eluting solvent for 30 min at room temperature. The developed plate was dried in normal atmosphere and spots visualized under visible light, UV: 254 nm and UV: 366 nm. The *R*_*f*_ (retention factor) values of separated compounds of the fraction and standards were calculated and compared.

### 2.7. Cospotting for Confirmation of Compounds

Equal amounts (40 *μ*L) of the sample (ethyl acetate extract), mixture of the sample with gallic acid, and only gallic acid were applied on a TLC plate (silica gel 60F_254_) (Merck, Germany) and developed in a previously saturated glass chamber (mobile phase of dichloromethane : ethyl formate : formic acid (5 : 5 : 0.2)) at room temperature. The developed plate was air-dried and sprayed with anisaldehyde/sulphuric acid mixture, heated (100°C), and visualized under visible light. The presence of umbelliferone was also confirmed as above using a mobile phase of toluene : ethyl acetate (6 : 4) at room temperature. The plate was air-dried and viewed under 366 nm.

### 2.8. High Performance Liquid Chromatography (HPLC) and LC UV/VIS Spectra

The homogenized samples of* S. cumini* decoction and the herbal drink were filtered through SEP-PAK C18 cartridge and the filtrate diluted (50%) with distilled water. HPLC analysis was performed using the HPLC system (Waters, USA) consisting of a pump controller (600 S) and a diode-array detector (DAD, 996 UV detector). Conditions for analytical HPLC used were Eclipse XDB C 18 column (5 *μ*m, 150 mm × 4.6 mm) (Agilent Technologies, USA) with a flow rate of 1.0 mL/min with 5 *μ*L of sample injected. Detection was carried out at 254 nm. Phenolic compounds in the decoction and the RTS herbal drink were analyzed using isocratic elution with 1% acetic acid and acetonitrile (80 : 20) solvent mixture as the mobile phase. The LC UV-VIS spectra were obtained and compared with the UV-VIS spectra of the pure compounds created using the Millennium Chromatographic Manager package.

## 3. Results and Discussion

In the present study an attempt was made to scientifically prove the efficacy of utilizing the decoction made from dried bark of* S. cumini* (madan, Sinhala) in the treatment of diabetes by confirming the presence of antidiabetic compounds with antioxidant and antiglycation potential. In addition, the presence of such compounds in the RTS drink made with the said decoction was also studied. The antioxidant and antiglycation potentials of the RTS drink have been proven earlier and the quantity of* S. cumini* decoction in the herbal drink is below the prescribed dose for diabetes in Ayurveda [11]. In the process of activity guided fractionation by sequential extraction, the hexane fraction had significantly low antioxidant and antiglycation activities while ethyl acetate and water fractions had higher activities. Since the ethyl acetate fraction showed high antioxidant and antiglycation activities [13] a preliminary separation of compounds on TLC was compared with compounds of proven antidiabetic properties. This illustrated the presence of gallic acid and umbelliferone in this fraction and hence the decoction. The retention factors (*R*_*f*_) confirmed the presence of gallic and umbelliferone in the ethyl acetate extract with some other compounds which were not identified (Table 1).

**Figure 1:**
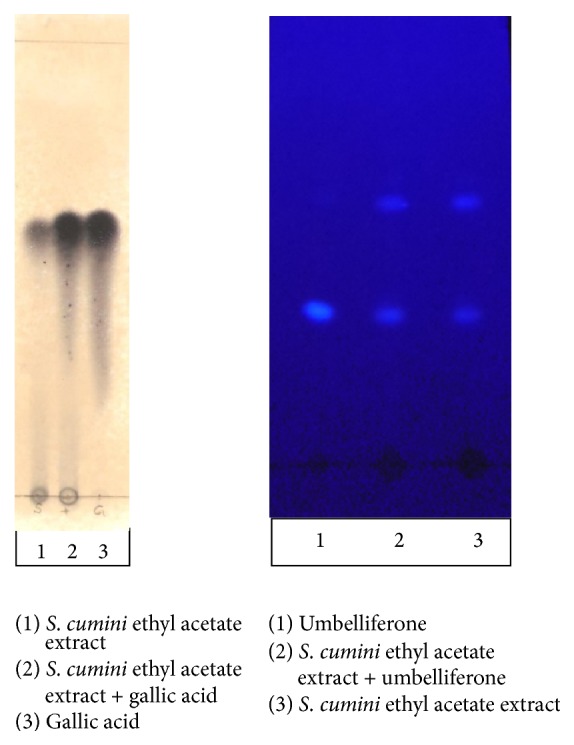
Co-TLC of ethyl acetate extract with gallic acid and umbelliferone (gallic acid, visible; umbelliferone, UV 366).

The presence of gallic acid and umbelliferone in the ethyl acetate fraction was further confirmed by TLC-cospotting (Figure 1) using different solvent systems.

Gallic acid (3,4,5-trihydroxybenzoic acid), a naturally occurring low molecular weight triphenolic compound, is a strong antioxidant and an efficient apoptosis inducing agent [15]. Several studies have proven the significant antidiabetic effects and cardioprotective effects of gallic acid. Gallic acid increases GLUT4 translocation and cellular glucose uptake [16] prevents diabetes-induced cardiac dysfunction and causes significant decrease in the malonaldehyde, an indicator of lipid peroxidation in STZ-diabetic rats [17], and protects RINm5F beta-cells from glucolipotoxicity by its antiapoptotic and insulin-secretagogue actions [18]. Umbelliferone (UMB), also known as 7-hydroxycoumarin, hydrangine, skimmetine, and beta-umbelliferone, belongs to the coumarin family. Umbelliferone is reported to have antioxidant properties and is the parent compound for a large number of natural products [19]. The reduction of blood glucose and better lipid profiles in diabetic rats given UMB indicate antidiabetic and antihyperlipidemic effects [20]. Ramesh et al. [21] had also proven the antidiabetic, antioxidant, and antihyperlipidemic properties of UMB. Thus the presence of gallic acid and umbelliferone which have ameliorative properties proves beyond doubt the scientific basis of using* S. cumini* decoction in the treatment of diabetes.

**Figure 2:**
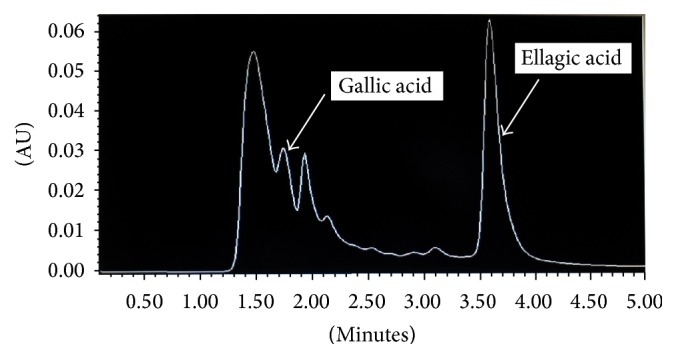
HPLC chromatogram of* S. cumini *RTS drink.

**Figure 3:**
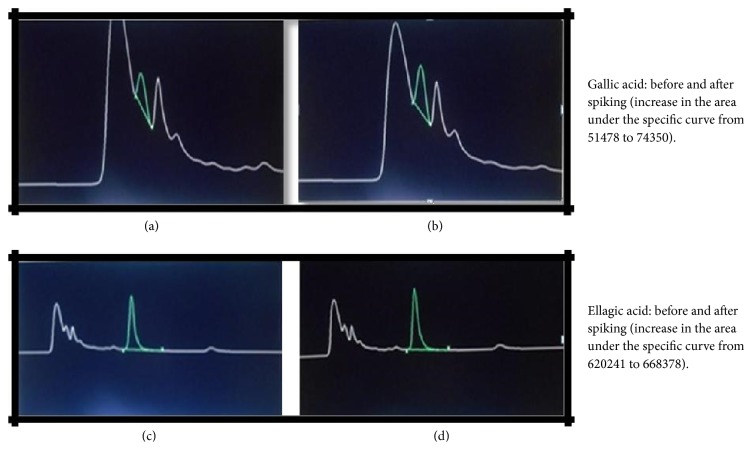
HPLC chromatograms of RTS drink before and after spiking with gallic and ellagic acids.

The next step involved confirming the presence of such compounds in the RTS drink made with the decoction taking into account the dose prescribed for diabetes [11]. The HPLC chromatogram of* S. cumini *RTS drink confirmed the presence of gallic and ellagic acids (Figure 2). The presence of gallic and ellagic acids was confirmed by spiking the RTS drink with 0.1 *μ*g of each acid (Figure 3). The approximate quantities of gallic and ellagic acids in the RTS drink were 5 mg/100 mL and 20 mg/100 mL, respectively.

**Figure 4:**
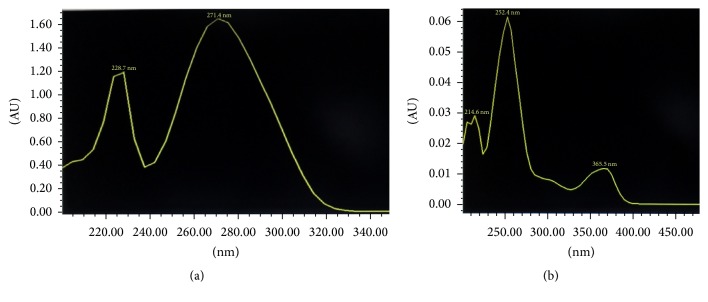
LC UV/VIS spectra of gallic acid (a) and ellagic acid (b) obtained from the RTS drink.

Further the LC UV/VIS spectra of the RTS drink when compared with the UV-VIS spectra of the pure compounds using the Millennium Chromatographic Manager package confirmed the presence of gallic and ellagic acids in the RTS drink (Figure 4).

Ellagic acid (EA) is a commonly found dietary polyphenol with antioxidant [22] and antiproliferative properties [23–25]. Muthenna et al. [26] reported on antiglycation properties of EA and its mechanism of action using an in vitro protein glycation system, where the inhibitory effect of EA on the formation of carboxymethyl lysine (CML) was shown to be prominent. Among the AGEs, CML is the most abundant compound in diabetes patients. The findings of their study confirmed the inhibition of glycosylated hemoglobin formation (HbA_1C_) in human blood under high glucose conditions and this signifies the physiological antiglycation potential of EA. They have proven the effectiveness of EA against loss of eye lens transparency through inhibition of AGEs in the lens organ culture system [26]. Thus the availability of EA in the ready to serve herbal drink could be highly contributory for the antiglycation effect and antioxidant effect of* S. cumini*. This further proves the therapeutic potential of* S. cumini* decoction in AGE mediated diabetes pathologies.

## 4. Conclusion

The study proves the presence of antidiabetic compounds such as gallic acid, umbelliferone, and ellagic acid in the decoction of* S. cumini* and the ready to serve drink. These plant phenolic compounds have proven antioxidant and antiglycation activities through which they can mediate their antidiabetic effects. Further the findings scientifically prove the efficacy of using* S. cumini* bark in herbal formulations used in the treatment of diabetes mellitus in Ayurveda medicine.

## Figures and Tables

**Table 1:** *R*_*f*_ values of standards and phenolic compounds in *S. cumini* ethyl acetate fraction.

Standard	*R*_*f*_ of the standards	*R*_*f*_ of the fraction
Gallic acid	0.07	0.075
Umbelliferone	0.51	0.51

Mobile phase: toluene : ethyl acetate (7 : 3).
